# Colorectal carcinoma with osseous metaplasia

**DOI:** 10.18632/oncotarget.18577

**Published:** 2017-06-20

**Authors:** Xibo Liu, Jinghong Xu, Lirong Chen

**Affiliations:** ^1^ Department of Pathology, The Second Affiliated Hospital Zhejiang University School of Medicine, Hangzhou 310009, China

**Keywords:** colorectal neoplasms, osseous metaplasia, immunohistochemistry, PCR, pathogenesis

## Abstract

Osseous metaplasia (OM) is rarely observed in colorectal cancer (incidence < 0.4% in rectal cancer), where it has a non-specific clinical presentation and unknown pathogenesis. Here, we report three cases of colorectal carcinoma with OM and propose a new hypothesis. All three patients (two males and one female) were Chinese and had different sites of colorectal carcinoma with OM: rectum, sigmoid colon, and appendix. The pathologic diagnoses were serrated adenocarcinoma; moderately to poorly differentiated adenocarcinoma with micropapillary carcinoma and cribriform comedo-type adenocarcinoma; and mucinous adenocarcinoma, respectively. Clinical follow-up showed that one patient died 5 months after surgery, but the others are alive after 68 months and 53 months. Immunohistochemistry revealed that CD44, MAPK, MDM2, OPN and PEDF were expressed by both tumor cells and stromal cells, while P53 was expressed only by tumor cells. KRAS/NRAS/BRAF genotyping revealed different KRAS mutations in each of the three cases, but the NRAS and BRAF exons were all wild-type. These findings suggest OM has no relation with NRAS and BRAF mutation, and it is uncertain whether there is a relationship between ossification and KRAS mutation. OPN, MAPK, MDM2, P53, PEDF and CD44 may act as osteogenic factors in colorectal cancer with OM.

## INTRODUCTION

Osseous metaplasia (OM), or heterotopic ossification, refers to the formation of bone at extraskeletal sites. Metastatic calcification and OM are observed under disease conditions in both normal tissues and tumors. OM is rare phenomenon in the gastrointestinal tract, but has occurred a few times in both benign and malignant tumors [1–3]. Indeed, OM in the gastrointestinal tract was first comprehensively described by Dukes in 1939 [4]. Since then, only a dozen cases have been reported [5]. Consequently, very little is known about the phenomenon. Here we present three cases of colorectal cancer with OM and provide insight into its pathological characteristics. We also review the relevant literature and discuss the possible pathogenesis of this particular neoplasm.

## RESULTS

The clinicopathological parameters and the KRAS, NRAS, BRAF and immunohistochemical findings from the three cases presented below are summarized in Table 1.

**Table 1:** Clinicopathological parameters

	Case 1	Case 2	Case 3
Age	76	64	69
Gender	Male	Male	Female
Symptom	Hematochezia	Chest pain and weight loss	no
Erythrocyte (1012/L)	2.97	4.07	3.04
CEA level (ng/ml)	54.8	61.43	19.3
OB	Positive	Positive	NA
Location	Rectum	Sigmoid colon	Appendix
Operation	Radical resection of rectal cancer	Sigmoid colon cancer resection	Appendectomy and tumor resection
Diagnosis	Serrated adenocarcinoma with mucus	Moderately to poorly differentiated adenocarcinoma with cribriform comedo-type adenocarcinoma and micropapillary carcinoma	Mucinous adenocarcinoma
Diameter (cm)	4.5	5	7
TNM	T2N1M0	T3N1M1	T4NxM0
Adjuvant chemotherapy	XELOX	FOLFOX and bevacizumab	5-Fu
Prognosis (month)	Alive, 68	Died, 5	Alive, 53
KRAS	4 exon	2 exon	2 exon
NRAS	WT	WT	WT
BRAF	WT	WT	WT
IHC Positive	CD44, MAPK, P53, MDM2, OPN, PEDF	CK20, MSH2, MSH6, MLH1, P53	CA199, CK20
IHC Negative	BRAF	CK7, PMS2, OCT3/4, Olig-2, BRAF	CA125, CA153, CK7, BRAF

NA, not available; WT, wild-type; IHC, immunohistochemistry.

### Case 1

A 76-year-old male complained of hematochezia, which had persisted for a month with increasing anal discomfort. The patient also had a three-year history of hypertension. Laboratory tests showed a reduced red blood cell count (2.97 × 10^12^/L), a positive fecal occult blood (FOB) test, and a raised CEA level (54.8 ng/ml). On colonoscopy, a neoplasm occupied the rectal cavity, and a biopsy showed adenoma with high-grade dysplasia. The patient underwent a laparoscopic radical resection of the rectal tumor with fashioning of an ileal stoma on March 23, 2011. Postoperative adjuvant chemotherapy (XELOX regimen) was then administered. Twenty-five months later, a right lung mass was found on a CT scan. The apicoposterior segment of the upper right lung and mediastinal lymph nodes were resected after a malignant tumor was diagnosed upon cytologic/biopsy examination. The patient was alive and disease-free 68 months after the first operation at follow-up in November 2016.

### Pathologic features

**Figure 1:**
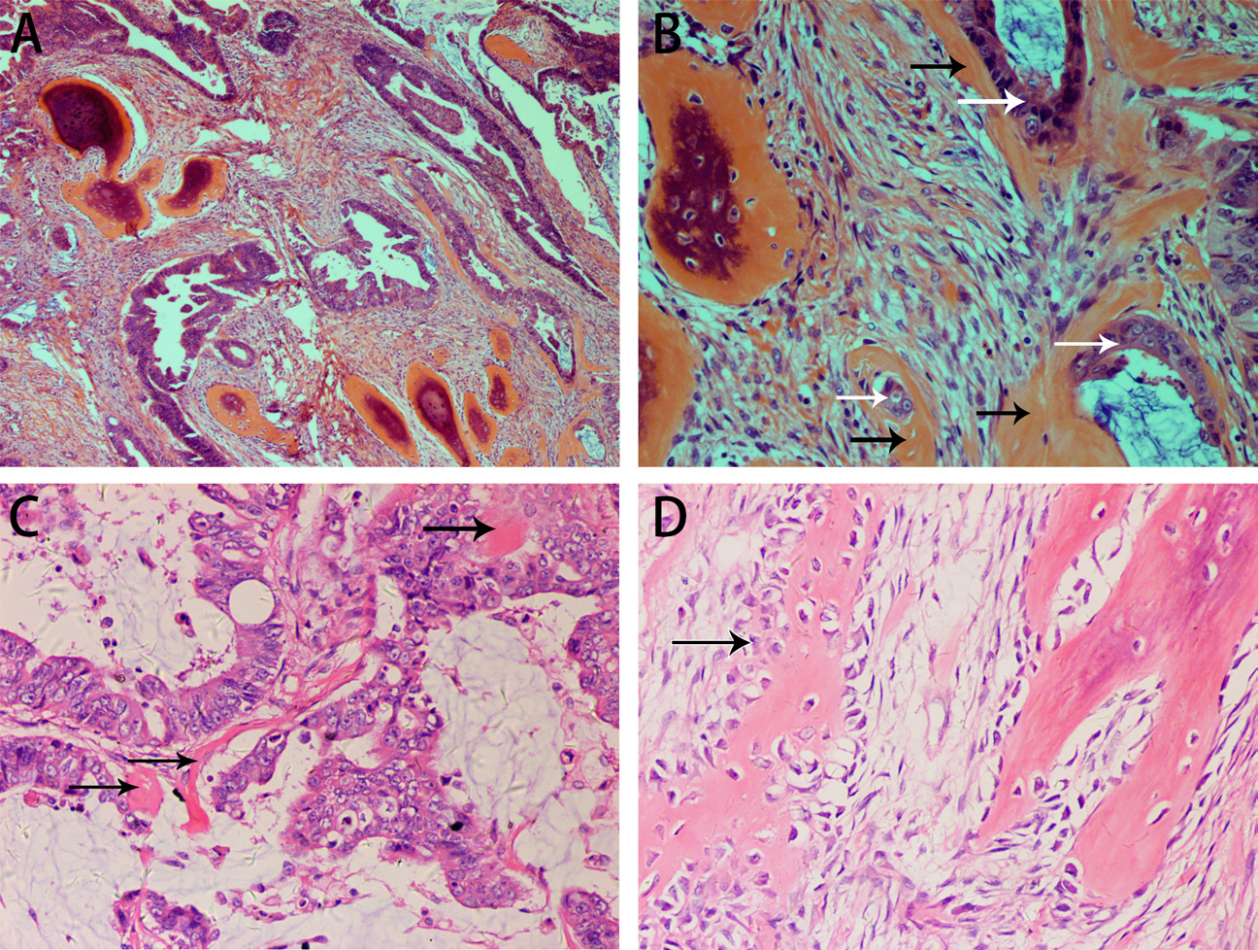
H&E staining showing the histopathological appearance of the rectal carcinoma with OM in case 1 Serrated adenocarcinoma with ossification in the stroma (**A**, **B**). Osteoid matrix closely surrounds tumor cells (B). Formation of a small osteoid matrix is adjacent to the tumor cells with surrounding mucin (**C**). Benign osseous metaplasia is rimmed with scattered osteoblasts. The boundary between osteoblasts and stroma cells is obscure (**D**). (black arrow: osteoblasts).

The resected rectum specimen contained a protuberant tumor measuring 4.5 × 4 × 4 cm. Histologically, the tumor showed a malignant epithelial tumor forming a predominantly serrated growth pattern with less than 50% mucinous area. The malignant cells had invaded the muscularis propria. Benign bone tissue was observed in the stroma. Laminated bone trabeculae of various thicknesses were scattered in the stroma, but neither necrosis nor hematopoietic fatty marrow was observed (Figure 1A). A diagnosis of serrated adenocarcinoma with OM was made. Some of the tumor cells were closely surrounded by osteoid matrix (Figure 1B and 1C). The margins between the osteoblasts and mesenchymal cells or fibroblasts were not clear. Osteoid, osteoblasts and osteocytes were scattered in and around osteoid matrix (Figure 1D). Pure calcification without ossification was not observed. Calcification and ossification were not found in the positive lymph nodes (3/7).

**Figure 2:**
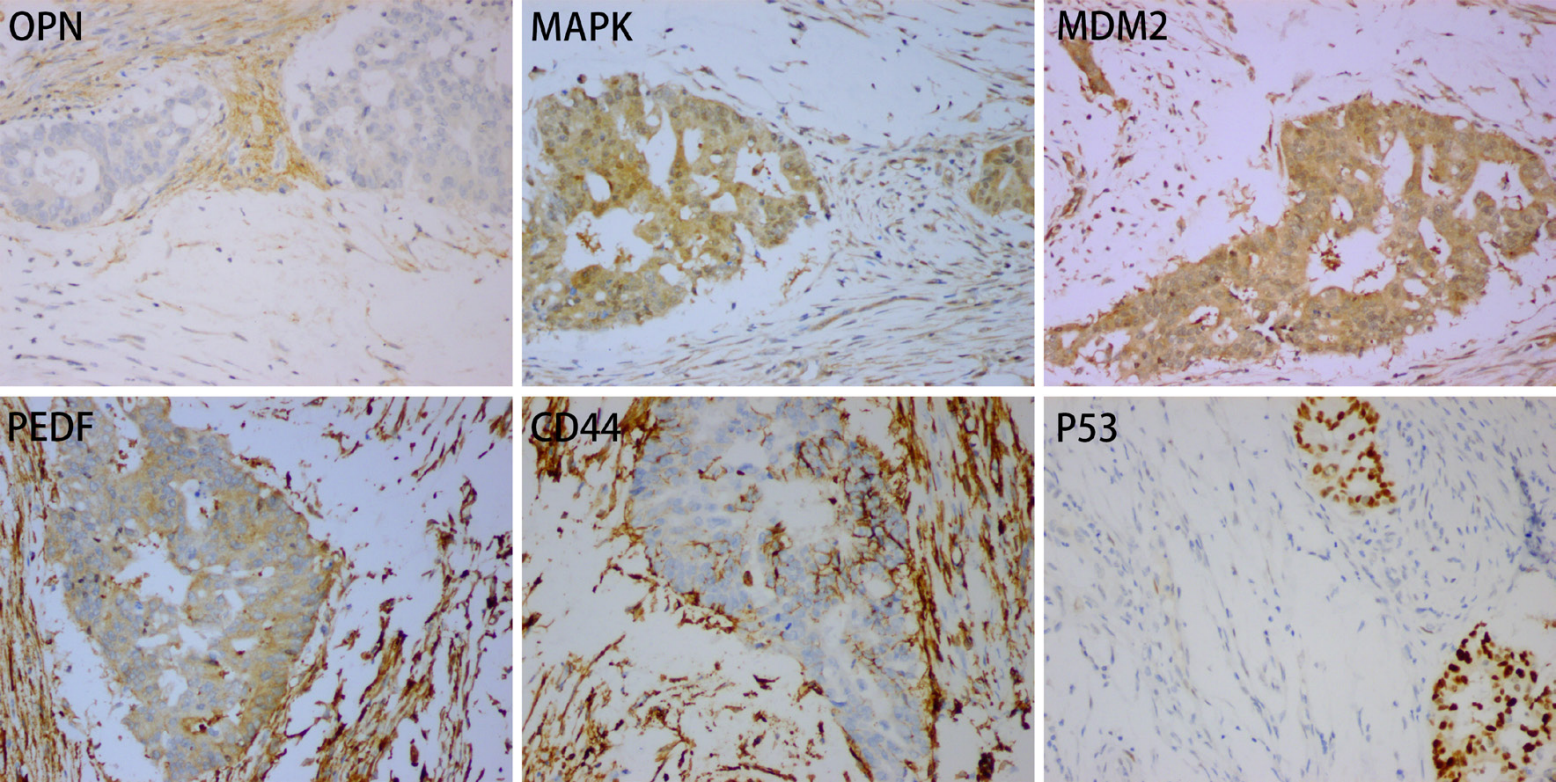
Positive expression for OPN, MAPK, MDM2, PDEF, CD44 and P53 in case 1

Immunohistochemically, the adenocarcinoma showed a weak positive reaction for osteopontin (OPN) and was moderately to strongly positive for MDM2, MAPK, PEDF, CD44 and P53. The stromal cells showed a moderate to strong positive reaction for OPN, CD44, MAPK, MDM2 and PEDF (Figure 2), and were negative for BRAF (Supplementary Figure 1). Examination for KRAS/NRAS/BRAF mutations revealed a KRAS exon 4 mutation (K117N, A146T, A146V or A146P), but neither NRAS nor BRAF gene mutation was found.

The right lung tumor measured 3 × 2.5 × 2.5 cm. The tumor cells were detected in both the fiber bronchoscope specimen and bronchoalveolar lavage fluid through cytological examination. The histological appearance was of a well-differentiated adenocarcinoma. Immunohistochemistry indicated a primary lung tumor with positive reactions for TTF-1 and CK7 and negative reactions for CK20, CDX2 and MUC-2 (Supplementary Figure 2).

### Case 2

A 64-year-old male with a month’s history of paroxysmal dull pain in the upper chest and a 5 kg weight loss over the previous 6 months was referred for further evaluation. The patient also had a 3-year history of hypertension. Physical examination revealed a tender, hard and poorly mobile mass in the left lower quadrant. The results of laboratory tests showed a reduced red blood cell count (4.07 × 10^12^/L), a positive FOB test, and a raised CEA level (61.43 ng/ml). A CT scan showed a space-occupying lesion in the middle lobe of the right lung and a nodule in the inferior lobe of the right lung. Colonoscopy revealed a mass causing lumenal stenosis. ECT showed no skeletal abnormality. Biopsies of both the colon and lung tumors revealed adenocarcinoma. Laparoscopic sigmoid colon resection was performed on November 26, 2014. FOLFOX and bevacizumab were used as postoperative adjuvant chemotherapy. Five months after the operation, the patient died after a tumor invaded the right main pulmonary artery and its proximal branches.

### Pathologic features

**Figure 3:**
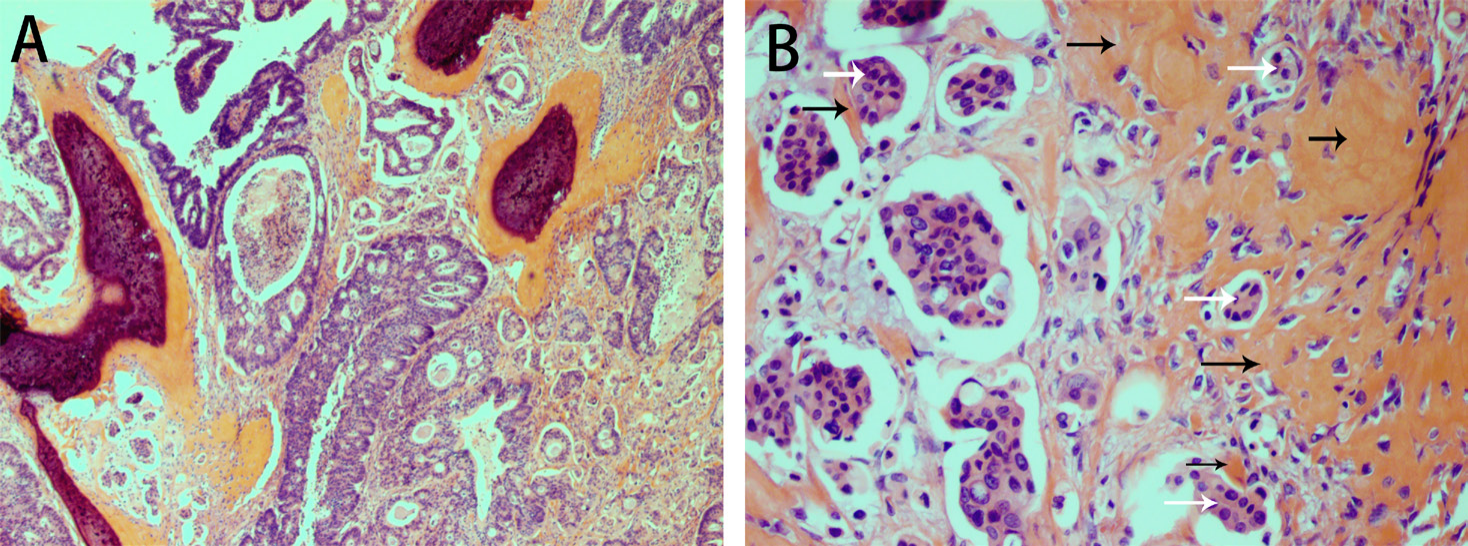
H&E staining showing osseous metaplasia in carcinoma of the sigmoid colon in case 2 Osseous metaplasia is present in the stroma. The tumor is a moderately to poorly differentiated adenocarcinoma with both cribriform comedo-type adenocarcinoma and micropapillary carcinoma (**A**). Osteoid matrix containing osteoblasts surrounds the tumor cells (**B**).

The sigmoid colonic tumor measured 5 cm in maximum diameter and was a moderately to poorly differentiated adenocarcinoma (micropapillary and cribriform comedo-type components) invading the subserosa. Focal calcification and ossification was observed in the stroma. Osteoid matrix containing osteoblasts closely surrounded the tumor cells (Figure 3). Necrosis was only present as part of the cribriform comedo-type adenocarcinoma. One positive (out of 18) lymph node was found but without OM.

Immunohistochemistry showed mismatch repair deficiency with reduced expression of MLH1 and loss of PMS2 expression. CK20 and P53 were positive, and CK7 and BRAF were negative (Supplementary Figure 3). KRAS/NRAS/BRAF gene mutation analysis revealed a KRAS exon 2 (G12S or G12D) mutation but neither NRAS nor BRAF gene mutation.

With regard to the lung tumor, fine needle aspiration cytology revealed the presence of malignant cells in the seventh group of lymph nodes. Immunocytochemical positivity for CK20 and CDX-2 and negativity for CK7, CK5/6, P63 and TTF-1 confirmed this to be metastatic colorectal cancer (Supplementary Figure 4).

### Case 3

A 69-year-old female with a 2-year history of elevated CEA levels (10.5 ng/ml–19.3 ng/ml) was admitted to our department. She also had a more than 1-year history of hypertension and atrial fibrillation. A CT scan indicated lesions with calcification in the appendix area and pelvic cavity, as well as multiple nodules in the mesentery, presumed to represent pseudomyxoma peritonei. An ultrasound scan showed mixed echogenicity in the right hypogastrium and suggested an appendiceal mucinous adenocarcinoma. The patient underwent an appendectomy and tumor resection on July 18, 2011 after frozen section histology confirmed the presence of malignancy. Adjuvant chemotherapy with 5-FU was administered. The patient was alive 53 months after the operation at follow-up in November 2016.

### Pathologic features

**Figure 4:**
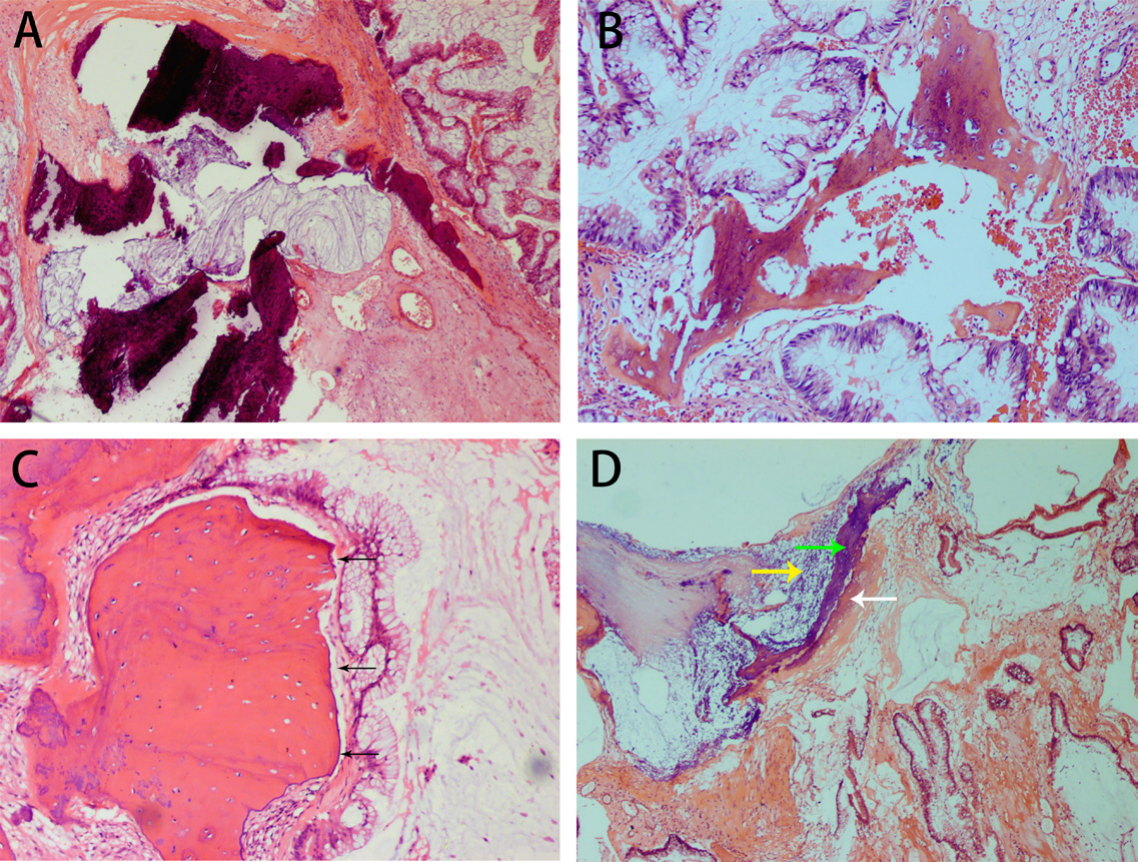
H&E staining showing the histopathological appearance of appendiceal mucinous adenocarcinoma with osseous metaplasia in case 3 Calcification and mucin are observed in the stroma (**A**). Well-differentiated carcinoma is present alongside osseous metaplasia. A small number of osteoblasts surround bone (**B**). A sharp boundary (black arrow) separates the osseous metaplasia from tumor cells, and few osteoblasts are seen in the margin of the osseous metaplasia (**C**). Calcium deposition and osteoid matrix are adjacent to the osseous metaplasia in a large proportion of the mucin (**D**). (white arrow: a formation of osseous matrix, yellow arrow: calcium deposition, green arrow: ossification).

The tumor located in the appendix measured 7 × 4 × 4 cm and was composed of well-differentiated adenocarcinoma with a large proportion of mucus. Sporadic calcification and ossification were observed. A sharp boundary was seen between the OM and tumor. Calcium deposition, osteoid matrix and adjacent ossification were found within the mucus (Figure 4).

The adenocarcinoma showed expression of CK20 and CA19-9 but no expression of CA125, CA153, CK7 or BRAF (Supplementary Figure 5). KRAS/NRAS/BRAF gene mutation analysis revealed a KRAS exon 2 mutation (G12C, G12R, G12V, G12A or G13C) but neither NRAS nor BRAF mutation.

## DISCUSSION

Although OM or heterotopic ossification is a particularly rare phenomenon in the gastrointestinal tract, it can occur in primary and metastatic tumors [5, 6]. There is no precise incidence data for OM in colorectal cancer. Dukes speculated that less than 0.4% of rectal cancers show ossification [4]. We experienced only three cases with OM among more than 2000 cases of colorectal cancer, which suggests an incidence of less than 0.15% in our hospital. Although this preliminary data shows a very low incidence of OM in colorectal cancer, the validity of this data needs confirmation with a larger number of cases. Ansari [5] reported a case of rectal carcinoma with ossification and reviewed the literature from 1923 to 1991. We will therefore summarize the literature written in English on colorectal carcinoma with OM from 1991 to 2016 (Table 2).

**Table 2:** Literature review of colorectal carcinoma with osseous metaplasia from 1991 to 2016

Case	Author	Year	Gender	Age	Location	Diagnosis	TNM	Treatment	Follow-up (month)
1	Noh, B. J.[13]	2016	F	76	Sigmoid colon	Moderately differentiated adenocarcinoma	NA	Anterior resection, twelve cycles of chemotherapy	Alive, 24
2	Smajda, S.[24]	2015	F	29	Rectum	Mucoid adenocarcinoma	pT3N2M1	Neoadjuvant chemotherapy, radiotherapy, complete excision of the tumor, adjuvant chemotherapy and stereotaxic radiotherapy	Died, 17
3	Badmos, K. B.[9]	2011	M	48	Colon	Mucinous adenocarcinoma	T3 NxMx	Resection of the rectosigmoid colon segment, adjuvant chemotherapy	NA
4	Al-Maghrabi, H.[25]	2005	F	90	Rectum	Well-differentiated adenocarcinoma	NA	Lower anterior resection	NA
5	Matsumoto, T.[26]	2004	M	67	Rectum	Well-differentiated adenocarcinoma	NA	Lower anterior resection	Died, 5
6	Kypson, A. P.[8]	2003	F	38	Rectum	Moderately differentiated adenocarcinoma	T3N1MX	Preoperative radiotherapy and 5-Fu, abdominoperineal resection, Adjuvant chemotherapy	NA
7	Imai, N.[14]	2001	F	50	Ascending colon	Moderately to poorly differentiated adenocarcinoma	NA	Hemicolectomy	Alive, 9
8	Alper, M.[27]	2000	F	56	Colon	Mucinous adenocarcinoma	NA	Colon resection	NA
9	Beauchamp, N. J.[28]	1997	M	64	Rectum	Moderately differentiated adenocarcinoma	T4N2Mx	Resection of the rectosigmoid colon segment	NA
10	Hui, Y.[29]	1995	F	66	Ascending colon	Adenocarcinoma	NA	Right colectomy	Alive, 24
11	Ansari, M. Q.[5]	1992	F	56	Rectum	Well-differentiated adenocarcinoma	NA	Preoperative radiotherapy, low anterior resection	NA
12	Lauwers, G. Y.[11]	1991	M	52	Left hemicolon	Moderately differentiated adenocarcinoma	NA	Left hemicolectomy with anterior rectal resection	NA

NA, not available.

The histological subtypes of our three cases were serrated adenocarcinoma, moderately to poorly differentiated adenocarcinoma with cribriform comedo-type adenocarcinoma and micropapillary carcinoma, and mucinous adenocarcinoma. Although one patient died 5 months after surgery, it is generally assumed that there is no direct relation between prognosis and the presence of OM in colorectal cancer [7, 8]. The exact pathogenesis of OM in colorectal carcinoma is unknown. However, bone-forming cells, protein matrix, cytokines and several growth factors are thought to be involved in the process [9]. Mucin, necrosis and desmoplastic stroma are also considered to be relevant factors [4, 10–12]. Among our three cases, two showed mucinous material and another contained necrosis. Van Patter speculated that calcium deposition plays an important role in the formation of heterotopic ossification [12]. Our observation of calcium deposition adjacent to heterotopic ossification in mucin would seem to support this hypothesis (Figure 4D), though our finding of calcification without ossification in one case demonstrates that simple calcification is not essential to the start of OM formation. As an important sign of OM, osteoid matrix was observed in both the stroma and around tumor cells, even clinging to the latter (Figures 1B, 1C and 3B). This phenomenon indicates that there is a close relation between OM and both stromal and tumor cells, which could be consistent with the epithelial-mesenchymal transition hypothesis proposed by Noh [13]. Based on the morphologies, we suggest different mechanisms may underlie OM formation in different histological subtypes of colorectal carcinoma.

Bone morphogenetic proteins (BMPs), which include more than 20 subtypes, are factors associated with bone formation [8, 14]. Expression of BMP2, BMP4, BMP5, BMP6, BMP9 and osteocalcin has been reported in colorectal cancer with heterotopic ossification [8, 13–15]. We used antibodies against OPN, MAPK, MDM2, P53, PEDF and CD44 to detect expression in rectal carcinoma with OM. We showed that tumor cells were positive for all of these biomarkers, and that there was also moderate to strong expression of OPN, MAPK, MDM2, PEDF and CD44 in the stroma cells (Figure 2).

Animal experiments revealed that early OPN expression may facilitate pre-osteoblastic proliferation and migration, while subsequent downregulation may be required for hydroxyapatite crystal formation [16]. As important signaling kinases for osteogenic differentiation, MAPKs (including ERK and JNK) regulate osteogenic differentiation through transcriptional regulation. This may provide an important signal for bone formation in a stiff bone environment [17, 18]. MDM2 positively affects osteocalcin promoter activity. Moreover, MDM2 may combine with P53 as part of a multiprotein complex that regulates osteocalcin gene expression [19]. PEDF is capable of inducing differentiation of precursor cells into mature osteoblasts [20]. PEDF is also capable of inhibiting osteoclast function via upregulation of osteoprotegerin in primary osteoblasts and osteoclast precursor cells [21]. This is noteworthy, as ossification involves not only the activation of osteogenic function, but also inhibition of osteoclastic function. CD44 has an inhibitory effect on osteoclastogenesis, depending on the microenvironment [22, 23]. Thus, osteogenesis is highly regulated process governed by multiple genes.

For the first time, we have detected KRAS mutations in colorectal carcinoma with OM. KRAS mutations were detected in all three cases, while all NRAS and BRAF codons tested were wild-type. In addition, the absence of BRAF expression was verified immunohistochemically (Supplementary Figures 1, 3 and 5). This strongly suggests the pathogenesis of OM has no relation to NRAS and BRAF mutation. It is uncertain whether there is a relationship between OM formation and KRAS mutation, as the mutated codon differed in each case. There are two possible explanations for this: 1) OM has no relation to KRAS mutation or 2) different KRAS mutations in different histological subtypes activate corresponding downstream signaling pathways, up or downregulating relevant proteins, ultimately leading to ossification.

In sum, we report three cases of colorectal carcinoma with OM. Our findings indicate there are several histological patterns of OM in colorectal cancer, and that OM has no relation to NRAS and BRAF mutation. However, it is uncertain whether there is a relationship between ossification and KRAS mutation. Moreover, OPN, MAPK, MDM2, P53, PEDF and CD44 may function as osteogenic factors in colorectal cancer with OM.

## MATERIALS AND METHODS

The clinical pathological parameters of all three cases of colorectal carcinoma with OM were selected from archives. Formalin-fixed paraffin-embedded tumor tissues were prepared for immunohistochemistry and fluorescence PCR. The samples of primary colorectal tumor were reacted with monoclonal antibodies against CD44, MAPK, MDM2, OPN, PEDF, P53, and BRAF in case 1; CK20, MSH2, MSH6, MLH1, P53, CK7, PMS2, and BRAF in case 2; and CA199, CK20, CA125, CA153, CK7, and BRAF in case 3.

Nucleic acids were extracted followed the manufacturer’s instructions (ADx-FF01, AmoyDx, Xiamen City, Fujian, China). Mutational analysis of KRAS (exon 2: G12S, G12D, G12C, G12R, G12V, G12A, G13C, G13D, exon 3: Q61L, Q61R, Q61H, exon 4: K117N, A146T, A146V, A146P), NRAS (exon 2: G12D, G12S, G13D, G13R, G12C, G12V, G12A, G13V, exon 3: Q61R, Q61K, Q61L, Q61H, exon 4: A146T), and BRAF (exon 15: V600E1, V600K, V600E2, V600R, V600D1, V600D2) was performed using fluorescence PCR with AmoyDx^®^ KRAS/NRAS/BRAF mutation detection kits (ADx-KN04-Mx, AmoyDx, Xiamen City, Fujian, China) and then evaluated using Mx Pro software (AmoyDx, Xiamen City, Fujian, China).

## SUPPLEMENTARY MATERIALS FIGURES



## Abbreviations

OM: osseous metaplasia
PCR: polymerase chain reaction
IHC: immunohistochemistry
FOB: fecal occult blood
CEA: carcino-embryonic antigen
CT: computed tomography
ECT: emission computed tomography
FNAC: fine needle aspiration cytology
5-Fu: fluorouracil
