# Supplementary material for: Colorectal carcinoma with osseous metaplasia
Source: Oncotarget. 2017 Jun 20;8(39):65407–13. doi: 10.18632/oncotarget.18577 (PMC5630340; doi:10.18632/oncotarget.18577)
Supplement: Supplementary file 1 [file oncotarget-08-65407-s001.pdf]

Colorectal carcinoma with osseous metaplasia

Supplementary Materials

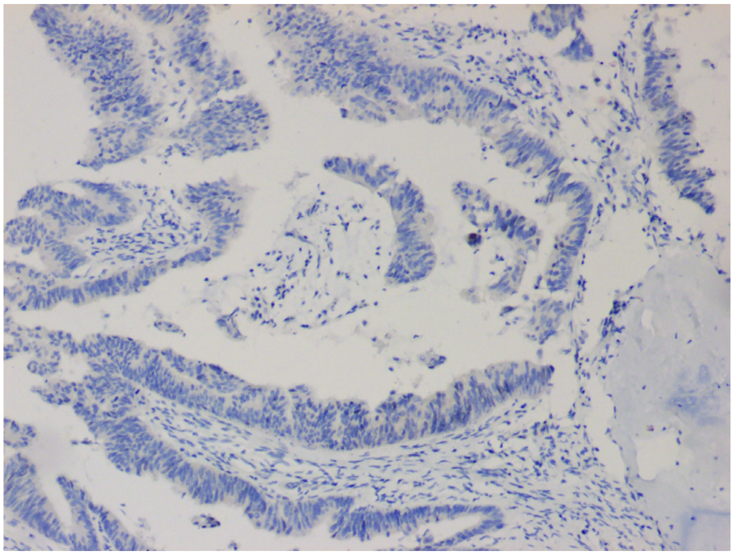

Supplementary Figure 1: Immunohistochemistry showing a lack of BRAF expression in case 1.

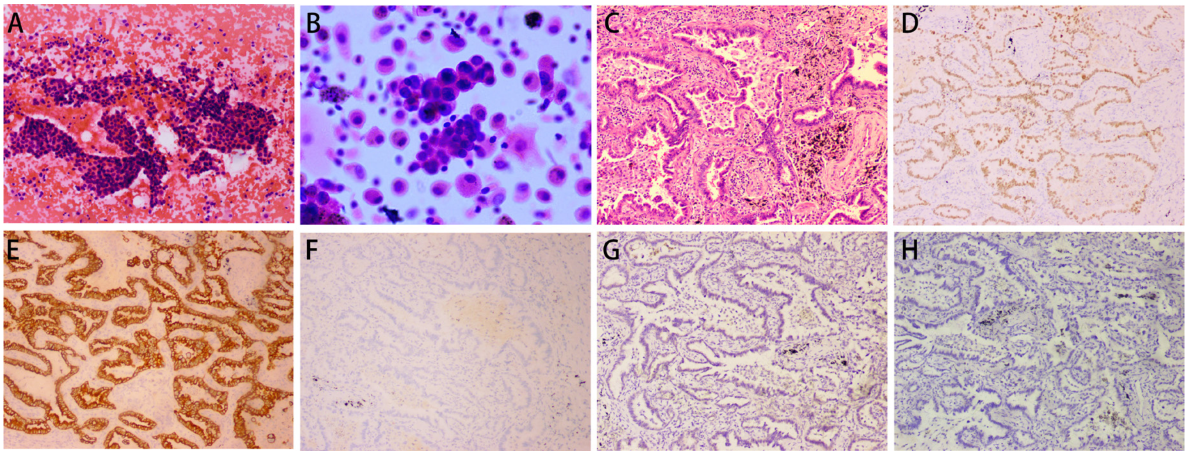

Supplementary Figure 2: Histopathological appearance and immunohistochemical analysis of lung cancer in case 1. Malignant cells were observed in both the fiber bronchoscope brush (**A**) and aspirated cytological specimens in pleural fluid (**B**). The histological appearance was of a well-differentiated adenocarcinoma of the lung (**C**). Immunohistochemical staining was positive for TTF-1 and CK7 (**D** and **E**) and negative for CK20, CDX2 and MUC-2 (**F**–**H**).

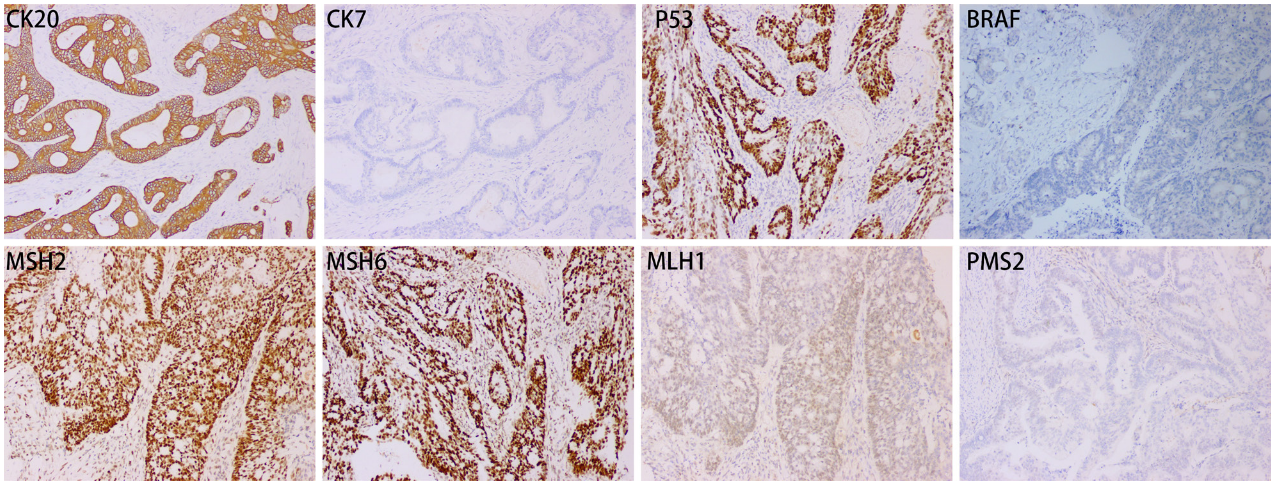

Supplementary Figure 3: Immunohistochemical positivity for CK20, P53, MSH2, MSH6, MLH1, and PMS2, and an absence of CK7, BRAF and PMS2 in case 2.

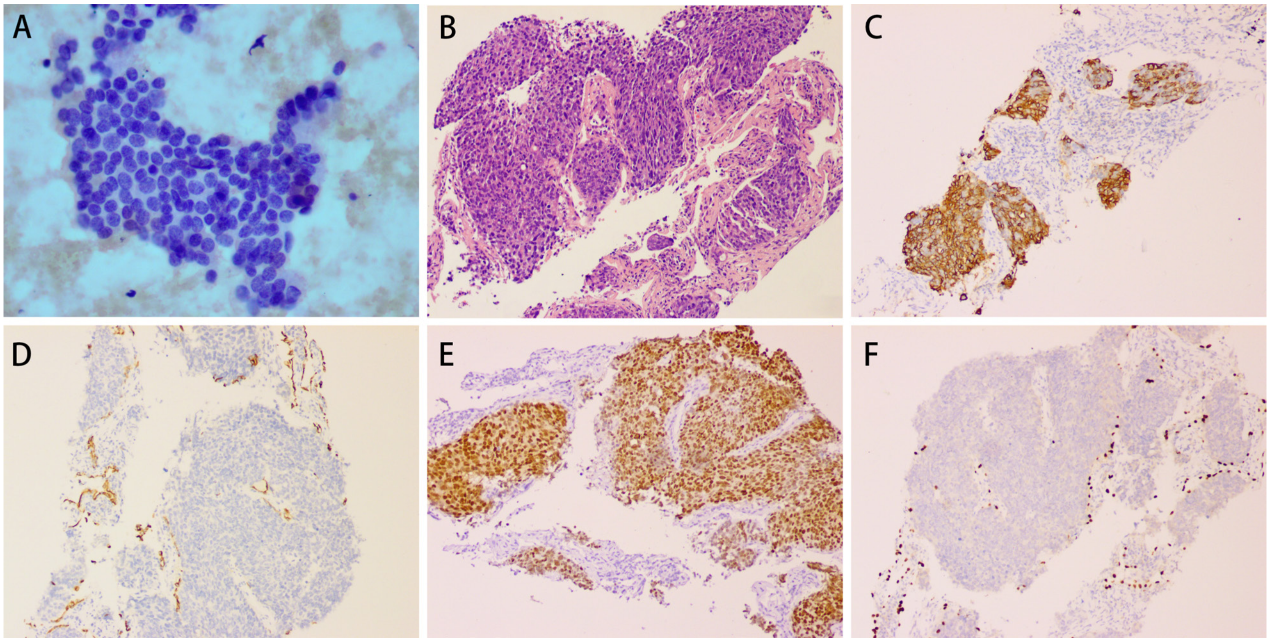

Supplementary Figure 4: Metastatic adenocarcinoma of the right lung in case 2. Malignant cells were found in cytology (**A**). H&E staining showed that tumor cells ranged from solid nests to infiltrative growth (**B**). Immunohistochemical staining was positive for CK20 and TTF-1 and negative for CK7 and CDX2 (**C**–**F**).

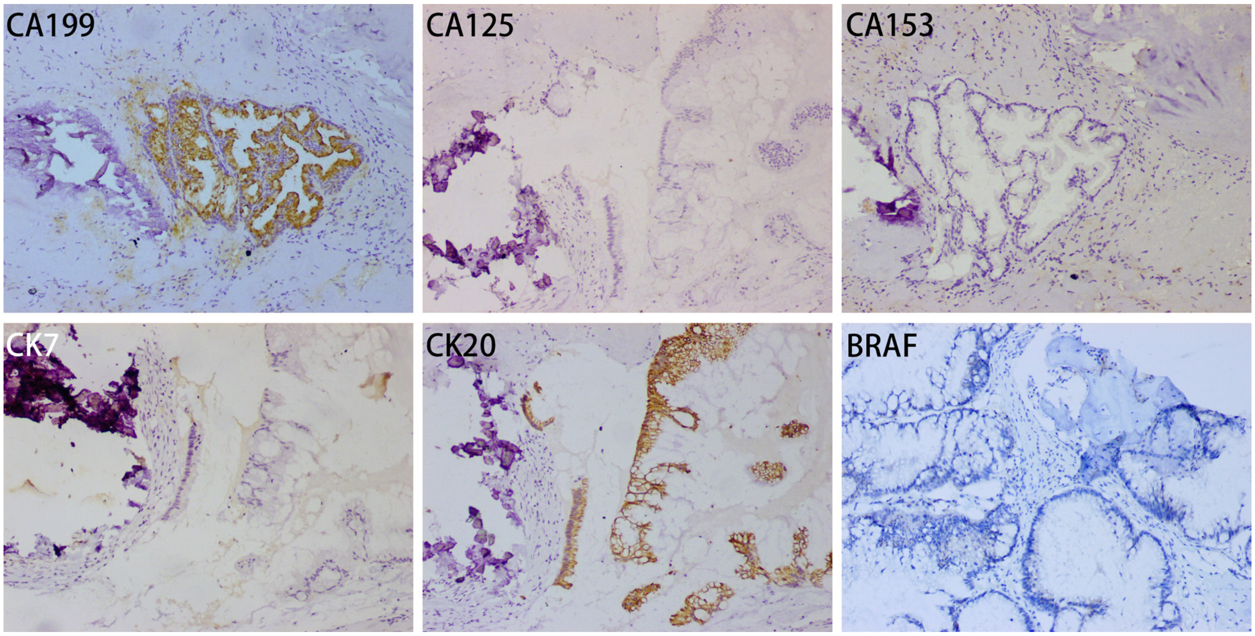

Supplementary Figure 5: Immunohistochemical staining was positive for CK20 and CA199 and negative for CA125, CA153, CK7 and BRAF in case 3.
